# The political challenge of realizing the right to health

**DOI:** 10.1002/gch2.1009

**Published:** 2015-10-23

**Authors:** Lauren Paremoer

**Affiliations:** ^1^ Department of Political Studies University of Cape Town Cape Town South Africa

## Abstract

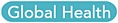

In recent years, global governance institutions have operationalized their commitment to the right to health by advocating for universal health coverage (UHC) – particularly in the Global South. UHC aims to develop health systems that are efficient, well staffed, and capable of providing affordable and appropriate medical care and essential medicines to rich and poor alike (World Health Organisation, 2014).

The political priority afforded to this goal is driven by an acknowledgement that people in wealthy and poor countries alike are priced out of the “market” for health care. This has caused concern because of the negative economic effects associated with inadequate access to care. Academic research and scholarly rhetoric often justifies UHC on the grounds that it will strengthen the economic position of households (particularly households living below or just above the poverty line) and contributes to the growth prospects of national economies (World Health Organisation Commission on Macroeconomics and Health, n.d.[Link]). UHC is thus framed as an investment in human capital: Healthier citizens are more productive citizens. They are more capable of investing in their own well‐being, and that of their dependents, through participation in the labor market rather than relying on public assistance.

Why is it problematic to justify UHC, and health promotion more generally, on the basis of its economic value, that is, on the basis of the contributions it makes to growing markets and improving economic productivity?

Research on the reconfiguration of welfare regimes in the Global North suggests that globalization is undermining their social, political, and economic foundations. These countries – much like their counterparts in the Global South – are experiencing a rise in unemployment, coupled with an increase in flexible and precarious work. Globalization has also been associated with an increase in human migration from the Global South to the Global North and within the Global South. As a result, states are simultaneously faced with increased demands for welfare from the unemployed and the working poor, an erosion of their tax base, and increasingly heterogeneous societies.

States are responding to these changes by adopting welfare policies that expand the influence of market actors and market logics on social welfare. Consequently, decisions about how to define and promote the public good are increasingly made in a decentralized fashion by private actors operating in households or markets rather than in democratic political institutions. For example, in many countries, access to public assistance is now contingent on welfare recipients' efforts to find employment (i.e., the shift from welfare to “workfare”) and pay for basic services, on private and public sector providers' ability to provide social services efficiently and cost effectively, and on the state's ability to efficiently coordinate interactions between citizen consumers and social services providers (Roche, 2002).

Advocacy for UHC reflects these political and normative shifts. It de‐emphasizes the importance of collective democratic decision‐making about how the health needs of populations should be addressed. Instead, public institutions are primarily responsible for solving a “technical” problem: financing health consumption for all. As public institutions become more focused on policing health financing, they reduce the services they provide and, in so doing, strengthen the market power of private players (Global Health Watch, 2014).

Private actors – philanthropic foundations and for‐profit providers of medical services, health insurance, and medicines – now routinely constrain the ability of governments to decide the terms on which the right to health should be advanced. Their influence is legitimated by the World Bank and IMF austerity policies that frame debt repayment and economic growth as the direct and primary responsibilities of democratic governments. Although governments in the Global South are particularly vulnerable to these pressures, similar pressures are present in the Global North – as demonstrated by private sector opposition to Obamacare (Kirsch, 2013) and patent law reform in South Africa (De Wet, 2014).

In contrast, these institutions frame the obligation to promote the social dimension of citizenship as something that can be outsourced to private actors and/or achieved through market logics. Additionally, the value of social policies is often justified in economic terms, for example, in terms of their ability to improve the productivity of worker citizens and the revenues of public and private sector service providers. This discourse de‐emphasizes the intrinsic value of formal and substantive equality and meaningful participation in collective decision‐making about the public good. Political institutions feature in it primarily as mechanisms that mediate the efficacy of investments in health care. Their significance is determined by their ability to maximize returns on investments in health (Jack and Lewis, 2009).

What are some of the consequences of advancing the right to health in this manner? Research shows that the shift from universalistic welfare regimes to regimes that target “especially vulnerable” or “especially deserving” populations undermines social solidarity by stigmatizing welfare recipients as people who violate the liberal ethos of contemporary welfare states. Vulnerable populations (e.g., non‐citizens, people of color, indigenous peoples, working class women, and able‐bodied unemployed people) are stigmatized as being reluctant to (or incapable of) succeeding in market societies on their own “merit” and as unfairly benefiting from welfare policies that advance their particularistic group interests rather than overall well‐being (Brown, 2003). The low social status of these groups obstructs their ability to access appropriate and effective medical care, even when it is available at no or little cost to patients (Bassett, 2015).

Globalization is a politically and socially mediated process. The harmful effects of globalization on social inclusion, and the limits of the policy responses to these dynamics, point to the urgent need for collective action and research aimed at addressing the dimensions of globalization that undermine the social determinants of health by privatizing, stigmatizing, and instrumentalizing the management of health – and in some cases, life itself. Collective action is needed to democratize decision‐making about health care at the local, national, and global levels in a meaningful way to foster social solidarity and address status inequalities that lead to disproportionate rates of illness and death among stigmatized social groups and to politicize the priority placed on economic growth, given its sometimes harmful effects on human and planetary health.

*Global Challenges* is a journal that welcomes scholarly contributions on these tough issues and insightful commentary that points toward strategies for addressing them.
